# Seasonal trends in the condition of nesting females of a solitary bee: wing wear, lipid content, and oocyte size

**DOI:** 10.7717/peerj.930

**Published:** 2015-05-07

**Authors:** Kevin M. O’Neill, Casey M. Delphia, Theresa L. Pitts-Singer

**Affiliations:** 1 Department of Land Resources and Environmental Sciences, Montana State University, Bozeman, MT, USA; 2 USDA-ARS Pollinating Insects Research Unit, Utah State University, Logan, UT, USA

**Keywords:** Megachile rotundata, Egg size, Reproduction, Megachilidae, Pollinators

## Abstract

During the nesting season, adult females of the solitary bee *Megachile rotundata* (F.) face considerable physical and energy demands that could include increasing wear and tear on their bodies and decreasing lipid reserves. Consequently, their reproductive performance may be affected not only by extrinsic factors (e.g., weather and floral resource availability), but intrinsic changes in their own bodies. Because of the potential fitness effects of seasonal changes in body condition, our objectives were to determine how wing wear, lipid reserves, and oocyte sizes vary during nesting seasons, beginning when females emerge as adults. As nesting progressed, females in two populations experienced a steady increase in wing wear, which is known to reduce foraging efficiency and increase risk of mortality in other bees. Soon after emergence, females exhibited sharp declines in lipid content which remained low for the remainder of the season. Newly-emerged females ingested pollen, an activity known to be correlated with the initiation of egg maturation in this species. Additionally, the early summer drop in lipid stores was correlated with an increase in the size of the oocytes carried. However, by ∼6 weeks after emergence, oocytes began to decrease in length and volume, perhaps due to nutrient deficiencies related to loss of stored lipids. Our results suggest management of *M. rotundata* should include rearing bees at temperatures that maximize stored lipid reserves in adults and timing bee release so that significant pollen resources are available for both adults and offspring.

## Introduction

The nesting season of the leafcutting bee *Megachile rotundata* (F.) (Hymenoptera: Megachilidae) is a period of intense physical activity, each female acting on her own to modify and provision a nest cavity (Pitts-Singer & Cane, 2011). During this time, females experience considerable energy demands and physical stresses from the large number of flights taken to collect pollen, nectar, and nesting materials. Soon after emerging as adults, females also begin to produce relatively large eggs (Richards, 1994), as is the case in other solitary, nest-provisioning Hymenoptera (Iwata, 1955; Iwata, 1960; O’Neill, 2001). Thus, we expect females to experience seasonal declines in both physical condition and the lipid reserves they carry over from earlier developmental stages (O’Neill et al., 2011).

Questions concerning reproductive performance of adult *M. rotundata* are relevant to their value as pollinators of alfalfa grown for seed production (Pitts-Singer & Cane, 2011). Following removal of bee cells from winter storage, *M. rotundata* are reared from prepupae to adults over 3–4 weeks before being released into field nest shelters containing thousands of potential nest sites. Rearing is timed so that cells are placed in fields as the alfalfa starts blooming and as adult females begin to emerge. Unlike honey bees and bumble bees, which produce new batches of foragers throughout the growing season, adults of solitary bees like *M. rotundata* emerge more synchronously. Even if a partial second generation appears later in the summer, it is the adult females from the overwintering generation that are present over the 4–6 weeks when most of the alfalfa flowers are pollinated (Strickler & Freitas, 1999; Bosch & Kemp, 2005). Thus, it is of interest to determine whether the condition of adult females changes seasonally, in ways that could affect the consistency of their performance as pollinators and as parents of the generation of bees that will be released during the following year’s growing season.

The effect of management strategies on the survival and development of the immature stages of *M. rotundata* has been studied during the summer nesting season (Pitts-Singer & James, 2008; Pitts-Singer, 2013b), during fall and winter storage of bee cells (Richards, Whitfield & Schaalje, 1987; Pitts-Singer & James, 2005; Pitts-Singer & James, 2009), and while bees are being reared prior to release into fields (Yocum et al., 2010; O’Neill et al., 2011). However, few studies have examined factors that could change seasonally and affect the fitness of adult females while they are nesting. First, wings of female solitary bees accumulate wear (Mueller & Wolf-Mueller, 1993; Wuellner, 1999; López-Uribe, Oi & Del Lama, 2008) that is known to affect the foraging success and reproductive performance of bees (Cartar, 1992; Higginson & Barnard, 2004; Foster & Cartar, 2010; Rehan & Richards, 2010). Second, lipid stores within fat bodies may decline, potentially influencing egg production, as they do in other insects (Arrese & Soulages, 2010). The importance of lipid stores is suggested by the fact that ∼20% of fresh weight of diapausing prepupae of *M. rotundata* consists of lipids. That value is greater than in most other insects, including honey bees (Buckner, Kemp & Bosch, 2004). Although much of the lipid in fat bodies may be metabolized during the pupal-to-adult transition, some females emerge with lipids comprising >25% of their dry weight (O’Neill et al., 2011). A question that remains, however, is how rapidly these stores are depleted in adults. Third, the decline in lipid stores, whenever it occurs, may also be correlated with a reduction in the size of developing oocytes, which peak in volume about five weeks after emergence (Richards, 1994). Of these three female qualities, only temporal changes in ovary condition have been quantified (Richards, 1994), and questions remain regarding changes in oocyte size.

Given the potential fitness effects of seasonal changes in female body condition, the objectives of this study were to determine how wing wear, body lipid reserves, and oocyte sizes vary across nesting seasons, beginning at the time when females appear as adults. Our study was conducted during two field seasons at one site in Montana (MT) and one season at a site in Utah (UT), where we collected bees weekly or bi-weekly following their release into alfalfa fields. We also recorded qualitative assessments of the pollen carried in the guts of females, pollen being the only significant source of protein in adult female diets. We discuss the implications of the results for reproductive strategies of solitary bees and for management of this widely-used pollinator.

## Materials and Methods

### Study sites and collection methods

We collected bees at two sites: (1) ∼3 km west of Laurel, MT (45°39′10.99″N, 108°49′39.36″W) (2011 and 2012) and (2) Lewiston, UT (44°55′31.73″N, 111°54′00.20″W) (2012). At both sites, cooperating growers released *M. rotundata* into nest shelters within commercial seed alfalfa fields. Each year, our first sample was taken within 1–2 days of the release, by using sweep nets to collect females entering and leaving nest entrances. Most, if not all, females were recently eclosed and in the early stages of nesting activities. We returned to the sites weekly (or bi-weekly for most samples in 2011 in MT) to obtain further samples in the same manner. We continued sampling until (1) populations declined and few females remained active or (2) the growers removed the nests from fields (see below). All samples were immediately placed in a cooler with ice and frozen later the same day to reduce the likelihood of females sustaining further wing wear or metabolizing lipids after capture. In 2012 in MT, a second set of sweep samples was collected at nest shelters on five dates and used for analysis of ovary condition and relative oocyte size. Females in these samples were immediately placed in Kahle’s solution upon return to the lab. We originally planned to sample through the time when numbers of females declined later in the summer, but the grower at the MT site removed bee shelters from the field earlier than usual because of the condition of the alfalfa.

### Quantification of wing wear

To quantify wing-wear, we used the index of wing wear (WW) developed for bees by Mueller & Wolf-Mueller (1993). For each bee, we used a stereomicroscope to record the number of nicks on the apical edge of each forewing, the relative size of the nicks, and the proportion of intact margin to determine WW as follows: 0 = a completely intact apical margin; 1 = 1–2 nicks on the apical margin; 2 = 3–10 nicks on the margin; 3 = some wing margin intact, though heavily serrated, with >10 nicks; 4 = completely serrated with no apical wing margin intact, but with excisions less than half the width of the distal submarginal cell; 5 = wing as described in 4, but with excisions more than half, but less than the entire width of the distal submarginal cell; and 6 = wing as described in 4, but with excisions greater than the width of the distal submarginal cell (Mueller & Wolf-Mueller, 1993). Like Mueller & Wolf-Mueller, we ignored large excisions present on one wing only, because they seemed not to be indicative of gradual wing wear. We evaluated the margins of the left and right forewing of each bee and scored them separately according to the WW classes described above, and then averaged the scores from the forewings to derive a single WW score for each bee. We tested the hypothesis that WW varied with date of collection using Kruskal–Wallis Tests, followed by Dunn’s test for multiple comparisons (Zar, 1999; SigmaPlot v. 11.0; Systat Softfware, Inc.).

### Quantification of body lipid content

We quantified body lipid content using the same bees for which we measured WW, following a method used previously for solitary bees (Richards & Packer, 1994; O’Neill et al., 2011) and wasps (Strohm, 2000). Bees were removed from the freezer and placed within separate glass vials in a 55 °C drying oven. After four days, a subset of 15 bees was removed and their dry masses were individually determined to the nearest 0.01 mg on a Sartorius TE64 balance (Sartorius, Goettingen, Germany). After returning them to the oven, we reweighed the same 15 bees 24 h later to determine if their body masses had stabilized; if not, they were again returned to the oven and the procedure was repeated daily until stable masses were obtained. At that time, all bees were weighed. To extract lipids from dried bees, we added 10 ml of petroleum ether to each vial as soon as the bees were removed from the oven and capped it. After 10 days, we decanted the ether and air-dried the vials containing the bees for one hour under a laboratory hood. The vials were then placed back into the oven until the masses again stabilized (∼72 h) before a post-extraction body mass was obtained. We subtracted post-extraction dry mass from the pre-extraction dry mass to estimate of the amount of lipid extracted. The result was then divided by the pre-extraction dry mass to obtain an estimate of the proportion of dry mass comprised of lipids (*P_L_*) for each bee. Each year, all bees collected during the summer were subjected to lipid analysis at the same time to avoid any variation that might be introduced by minor variations in handling technique or conditions in the lab.

Using one-way analyses of variance (ANOVA; SigmaPlot v. 11.0, Systat Softfware, Inc.), we tested the hypothesis that the *P_L_* varied with (1) collection date and (2) WW. Before analysis, *P_L_* values were transformed as the arcsine of the square root of *P_L_* (Zar, 1999). Pairwise comparisons were conducted using Student–Newman–Keuls (SNK) tests. We compared proportions of females with *P_L_* values ≥0.10 and ≤0.05, contrasting the first collection date each year with all other collection dates combined using Fisher Exact Tests.

### Quantification of ovary condition and oocyte size

The females stored in Kahle’s solution were dissected under ethanol so we could measure oocytes using a stereomicroscope equipped with an ocular micrometer. We dissected from 26 to 32 females collected on each of five dates in 2012 (total *N* = 145). To reduce the likelihood of having second generation females included in the 3 August sample, we dissected only bees with WW ≥1 for that date. For each female, we recorded: (1) head width (HW; to the nearest 0.5 mm), as an indicator of overall body size, (2) WW as described above, but for the right forewing only (the latter was deemed sufficient because we had determined in the other wing wear analysis that WW values for the two wings on each female differed by ≤1 in >97% of females), (3) length (L) and diameter (D, at the midpoint of its long axis) of each of the three longest oocytes (if the oocyte was somewhat flattened by being pressed against the inner wall of the exoskeleton, we recorded the midpoint value between the greatest and smallest width), and (4) whether the largest oocyte was allantoid (i.e., sausage-shaped, or a cylinder with hemispherical ends), as is typical of mature oocytes (Iwata, 1955). For allantoid basal oocytes (i.e., those next in line for oviposition and the largest oocyte in each female), we estimated their volume (*V* in mm^3^) as *V* = (4/3)(*πr*^3^) + (*πr*^2^)(*L* − 2*r*), where *r* = *D*/2. Other oocytes approximated a prolate spheroid in shape, so we estimated their volume as *V* = (4/3)(*π*)(*r*^2^)(*L*/2).

The mean head widths of females dissected did not differ significantly among the five sample dates (one-way ANOVA: *F* = 1.61; df = 4, 140; *P* = 0.17). Nevertheless, to test the hypothesis that oocyte size variables changed across the nesting season independent of body size, we standardized oocyte variables across head widths by calculating the ratios of four oocyte size variables to HW. The four variables were the maximum length (*L*_basal_) and volume (*V*_basal_) of the basal oocyte and the summed maximum lengths (*L*_total_) and summed volumes (*V*_total_) of the three measured oocytes. We then used Kruskal–Wallis Tests to compare ratios among dates (with Dunn’s Test for comparisons among pairs of dates; *α* = 0.05).

To test the hypothesis that oocyte variables differed among females with different degrees of wing wear, we began by regressing *L*_basal_, *L*_total_, *V*_basal_, and *V*_total_ on HW. We used the resulting linear regression equations to determine which females had negative residuals (i.e., smaller than expected oocytes) and positive residuals relative to predicted values from the regressions. We used Mann–Whitney Tests to determine if females with negative residuals had WW values different from those with positive residuals. We also correlated WW with the four oocyte variables using Spearman Rank Correlation tests.

### Observations of pollen loads in guts

For each female dissected, we recorded whether their crops and midguts were mostly full of pollen, as opposed to having a few pollen grains. To determine whether most of the pollen was likely from alfalfa plants, we dissected ten randomly chosen females each from the 22 June and 3 August samples; these females were not included in the oocyte analyses. We chose those two days because our previous research showed that females collect mainly alfalfa pollen early in the nesting season, but often add significant amounts of pollen from other plant species later in the nesting season (O’Neill & O’Neill, 2010). Thus, we expected any changes that might occur in pollen loads in guts would be detected by examining females from the first and last collection dates of the year. We used a micropipette to remove a sample of pollen from their guts and examined the pollen using 10x and 40x power under a compound microscope. One hundred pollen grains counted along a transect on the slide were scored as being either alfalfa or non-alfalfa pollen based on comparison to reference collections that we made for previous studies (O’Neill et al., 2004; O’Neill & O’Neill, 2010).

## Results

### Seasonal trends in wing wear

**Figure 1:**
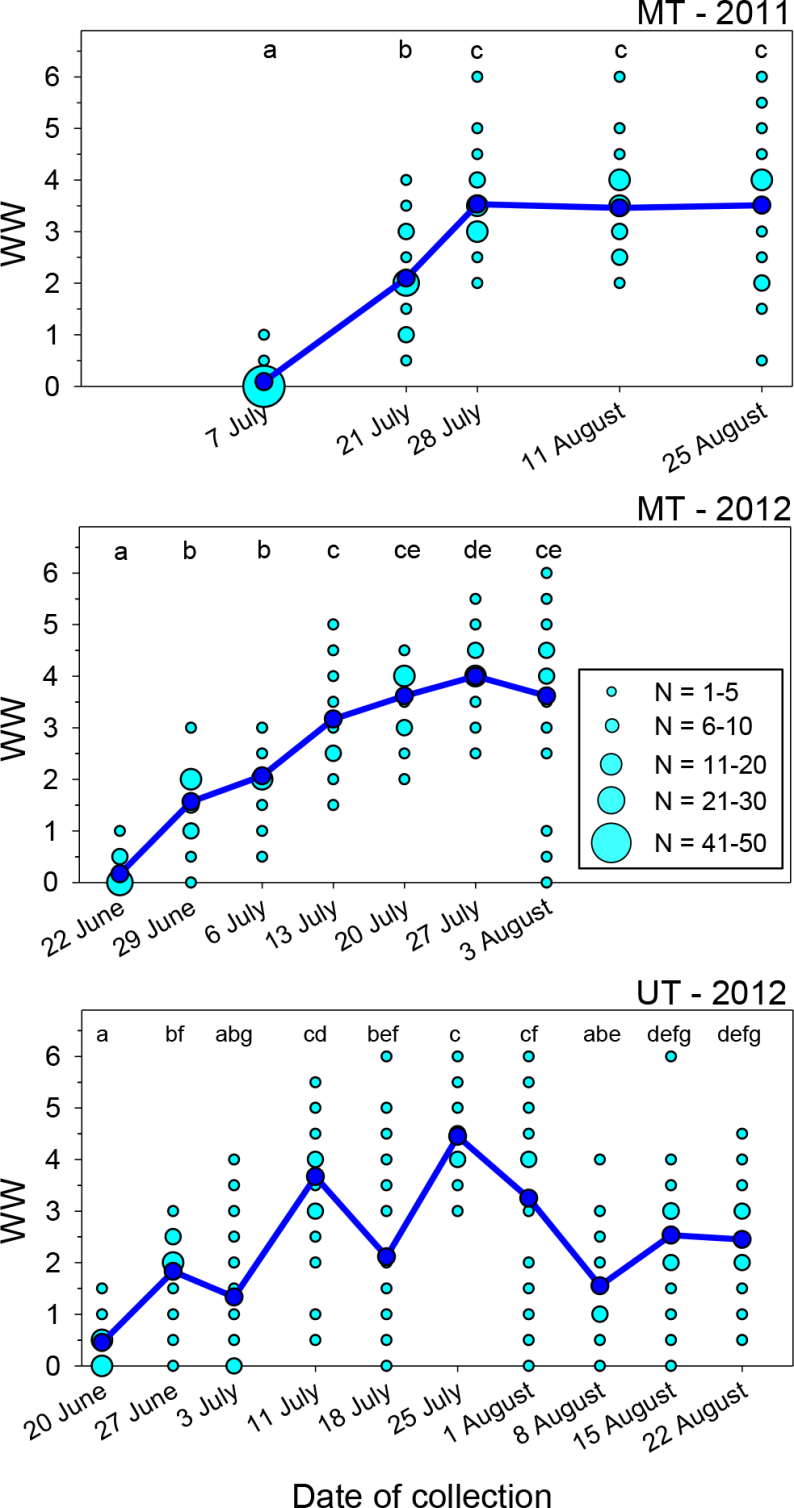
Seasonal changes in the index of wing wear (WW). Light blue circles represent raw data, dark blue circles and solid lines represent the mean values for each date. Means associated with different letters are significantly different at *P* < 0.05 (Dunn’s Test). Total sample sizes for each date were *N* = 50 (MT 2011) and *N* = 30 (MT 2012 and UT 2012).

Tattering of the forewings of *M. rotundata* occurs mainly along the apical margins where they are unsupported by bordering wing veins beyond the distal terminus of the costal vein. Wing wear varied significantly over the course of each nesting season at both locales (Fig. 1; Kruskal–Wallis Tests, *P* < 0.001 for each of the three sets of samples), but there was considerable variation among females collected after the first sampling dates. After a steady rise in mean WW early in the nesting season, it reached a plateau in both years at the MT site and declined at the UT site. Most bees collected on the first date each year exhibited WW values of ≤0.5, 96% for MT in 2011, 97% for MT in 2012, and 77% for UT in 2012.

In the two sets of MT samples, mean WW rose during the first 3–4 weeks following release. During the last three sampling dates combined, WW values were ≥4.0 in 43% of females in 2011 and 63% in 2012. For the UT bees, mean WW increased from <0.5 on the first collection date to 3.7 about three weeks following bee release, with 57% of WW values at that time being ≥4.0. However, mean WW then declined to 2.1 on 18 July (JD = 200), with 30% of the values being ≤0.5 and just 12% being ≥4.0; as discussed later, this likely occurred because of an influx of a second generation of bees into the population. The following week (JD = 207), the mean rose to its seasonal peak of 4.5, with all values exceeding 2.5 and 83% being ≥4.0. During the last three dates, the mean again declined to ∼1.5–2.5.

Wing wear was generally symmetrical between the left and right forewings. Among the 748 females examined, scores for the two wings were identical in 61.5% of females and differed by just one in another 35.7% of females. Wing wear scores never differed by more than two in any one female.

### Seasonal trends in lipid content

**Figure 2:**
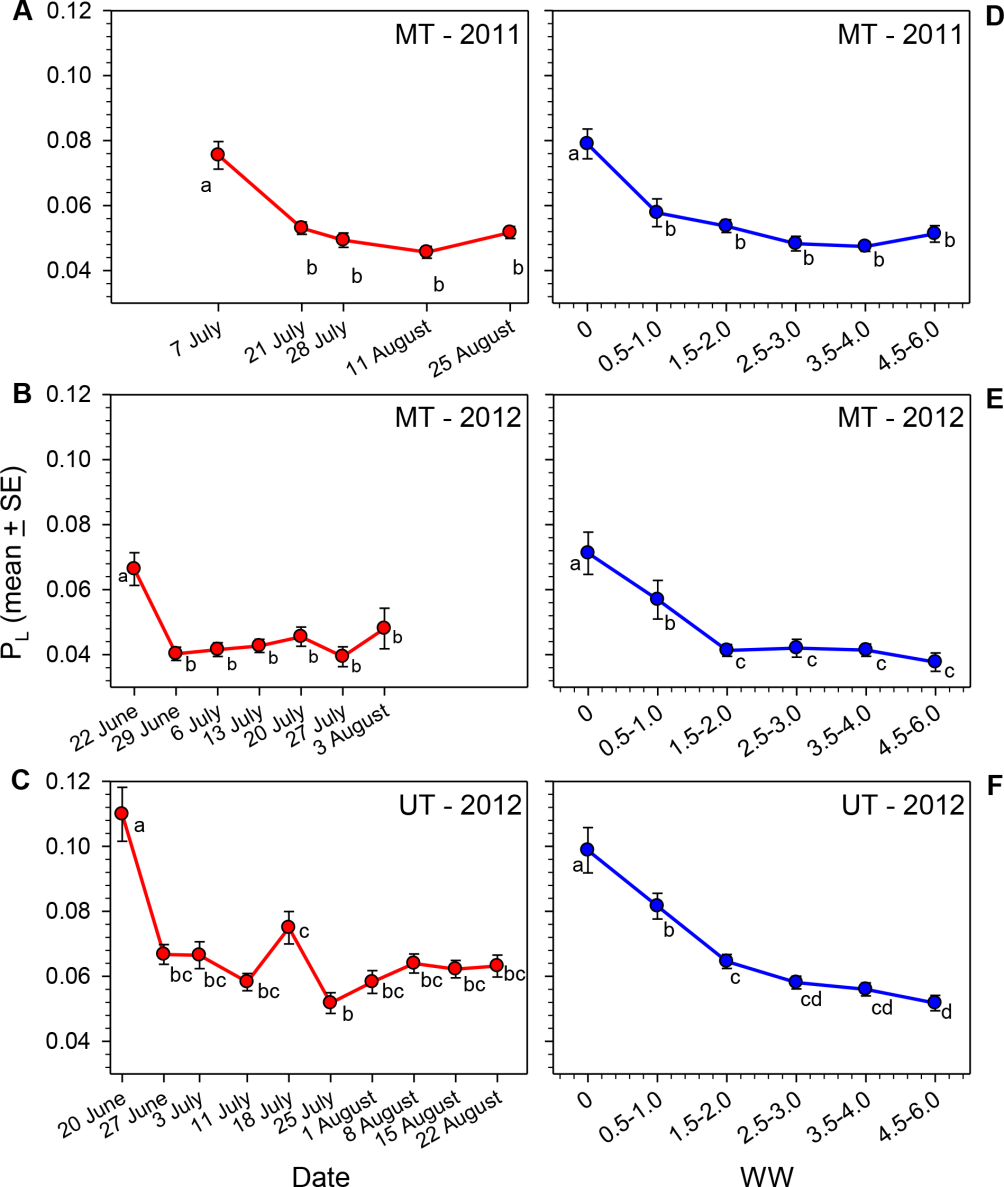
Seasonal changes in the relationship of *P_L_* for female *M. rotundata* to date of collection (A–C; red lines) and WW (D–F; blue lines). Means associated with different letters are significantly different at *P* < 0.05 (SNK Test).

The lipid content of females varied significantly over the course of the nesting season in all three sets of samples (Table 1, Figs. 2A–2C). Body lipids declined after field release, the initial decrease in mean *P_L_* ranging from 30 to 39% in the three sets of samples. The *P_L_* values then remained relatively constant for the remainder of each summer, with the exception of the brief increase in the 18 July (JD = 200) sample in Utah in 2012 (i.e., the same date on which WW values declined). The proportions of females with *P_L_* ≥ 0.10 on the first collection date was greater than the proportions for all other dates combined (Table 2). Conversely, the proportion of females on the first collection date with *P_L_* ≤ 0.05 was smaller than the proportions on all other dates combined.

**Table 1:** One-way analyses of variance for the relationship of *P_L_* to date of collection and WW for *M. rotundata* females.

Site	Year	*F*	df	Probability	Percent decline in mean *P_L_* from:	
***P***_***L***_ as function of date^*^					**First to second collection date**	**First to final collection date**
MT	2011	17.80	4, 245	*P* < 0.001	29.7%	31.5%
	2012	5.58	6, 203	*P* < 0.001	39.2%	27.5%
UT	2012	12.81	9, 290	*P* < 0.001	39.2%	42.5%
***P***_***L***_ as function of WW^*^					**WW = 0 to WW = 0.5–1.0**	**WW = 0 to WW = 4.5–6.0**
MT	2011	17.31	5, 244	*P* < 0.001	26.8%	35.1%
	2012	10.53	5, 204	*P* < 0.001	20.1%	47.5%
UT	2012	24.33	5, 294	*P* < 0.001	17.4%	47.5%

**Notes.**

* All analyses were done with arcsine-transformed *P_L_* values.

**Table 2:** Proportions of females with *P_L_* values ≥0.10 and ≤0.05, comparing the first collection date each year with all other collection dates combined.

Site	Year	Proportion females with *P_L_* ≥ 0.10		Fisher exact test probability	Proportion females with *P_L_* ≤ 0.05		Fisher exact test probability
		First collection date	All other collection dates		First collection date	All other collection dates	
MT	2011	0.260	0.005	*P* < 0.001	0.300	0.525	*P* < 0.01
	2012	0.166	0.011	*P* < 0.001	0.233	0.761	*P* < 0.001
UT	2012	0.566	0.048	*P* < 0.001	0.033	0.265	*P* < 0.001

Given the great variation in female WW values on most dates, it seems clear that date of collection is not an accurate estimate of females’ physiological ages (i.e., the cumulative amount of physiological/behavioral activity in their adult lives that is reflected in the degree of wing wear); this is especially the case later in the season when some second generation females emerge. In each sample *P_L_*, varied significantly across WW categories (Kruskal–Wallis Tests, *P* < 0.001 for each site/date combination; Figs. 2D–2F), with the decline in *P_L_* across WW classes, being more gradual than that observed across dates. Females with WW values ≥4.5 had mean *P_L_* values about 35% (MT 2011) to 48% (MT and UT 2012) that of females with WW values of zero (Table 1).

### Seasonal trends in oocyte size

In dissected females, the crop occupied most of the anterior half of the metasoma. The ovaries shared the limited space in the posterior half with other organs. Particularly when the crop was inflated with nectar/pollen, the interior cavity of the metasoma was often so crowded that larger oocytes were pressed against the inner surface of the integument and sometimes had indentations conforming to boundaries between metasomal segments. As oocytes matured and moved towards the position of being basal oocytes, they typically changed from being prolate spheroid-shaped to allantoid. Just 50% of the 30 females dissected from the 22 June sample had allantoid basal oocytes. Over the next three dates combined, 99% (*N* = 85) of females had at least one allantoid oocyte, but the percentage declined to 73% by 3 August (*N* = 30).

**Figure 3:**
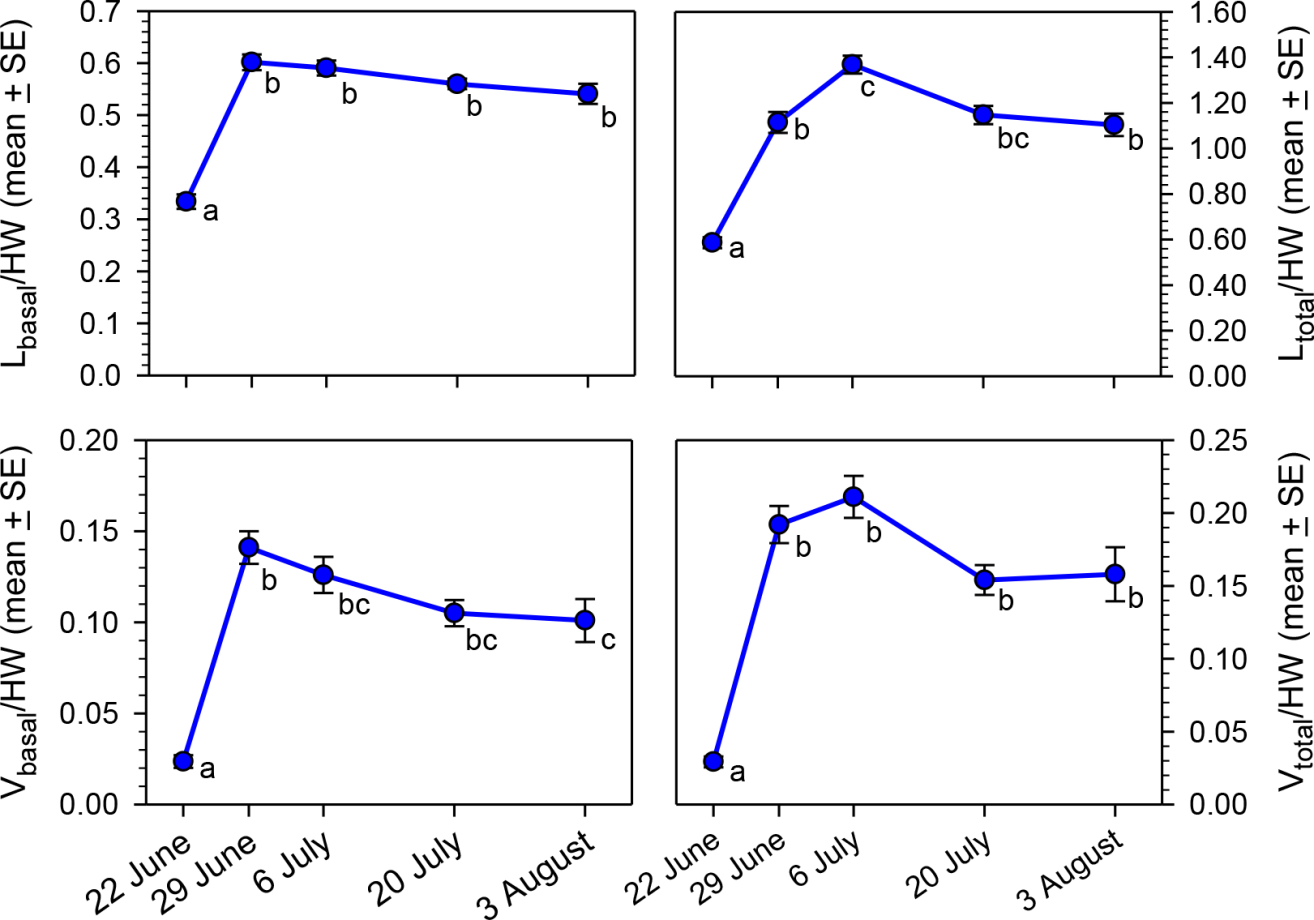
Seasonal changes in body size-adjusted oocyte size variables by sampling date for bees collected in MT in 2012. Means associated with different letters are significantly different at *P* < 0.05 (Dunn’s Test). Samples sizes for each date were *N* = 30 for 22 June, *N* = 26 for 29 June, *N* = 32 for 6 July, *N* = 27 for 20 July, and *N* = 30 for 3 August.

Our primary objective was to determine if oocyte size variables (*L*_basal_, *L*_total_, *V*_basal_, and *V*_total_) changed as the nesting season progressed, but analysis is complicated by the correlation of body size with oocyte size (O’Neill, Delphia & O’Neill, 2014). To remove the confounding effect of body size, we calculated ratios of each of the four oocyte variables to head width. All four ratios varied significantly across the five sampling dates (Fig. 3; Kruskal–Wallis Tests, *P* < 0.001 for each of the four variables), primarily because of the low ratios for 22 June. However, the *L*_total_/HW and *V*_basal_/HW ratios rose at the beginning of the nesting season, but then declined significantly by early August.

**Figure 4:**
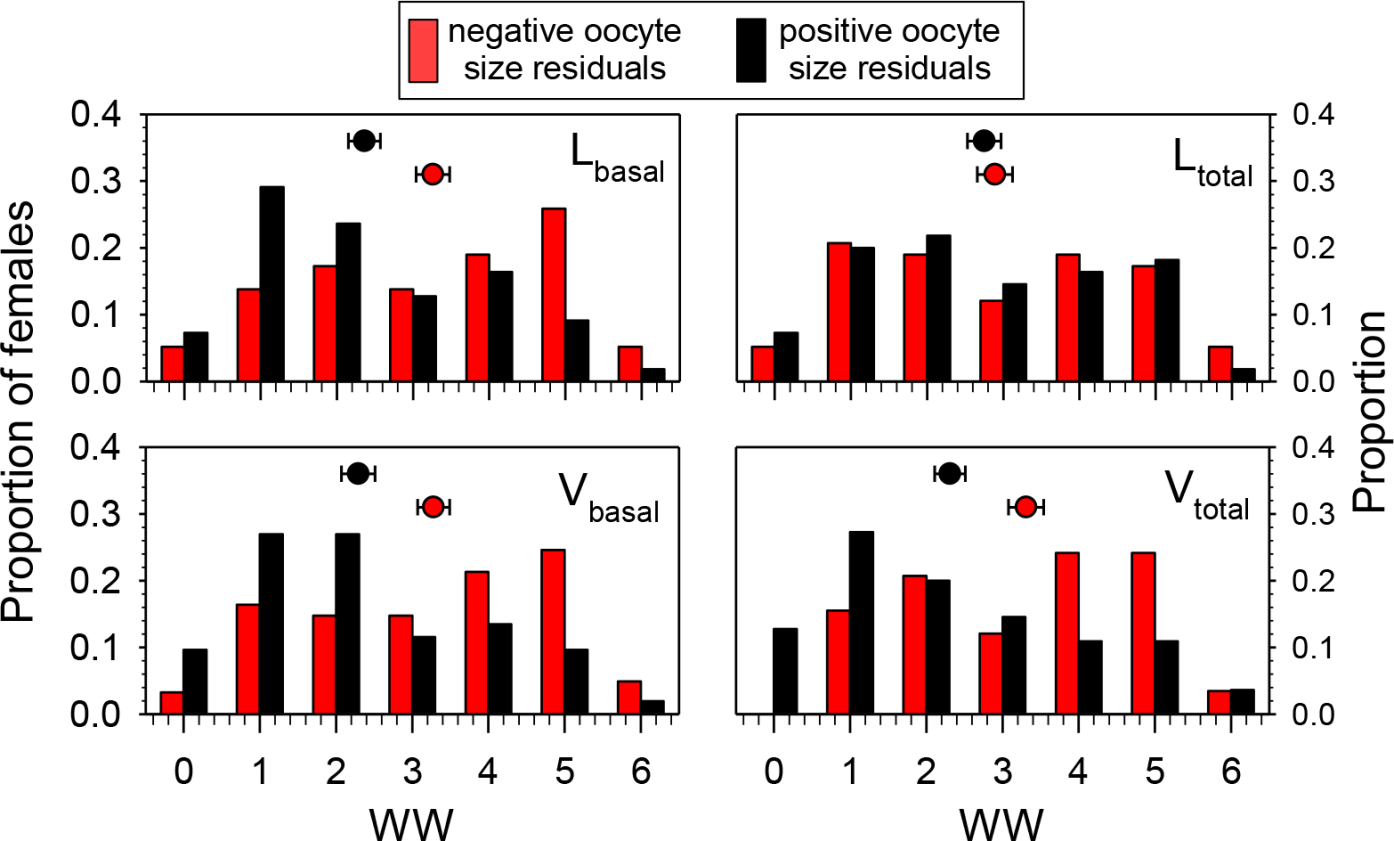
Proportions of females in different wing wear index (WW) categories with either negative or positive residuals for the regressions of *L*_basal_, *L*_total_, *V*_basal_, and *V*_total_ (all transformed as the square root of the value + 0.5) on head width (HW). Females with negative residuals had smaller than expected oocyte sizes based on the HW-oocyte size linear regression equation. The regression equations for oocyte variable (square-root transformed) on HW were: (1) *L*_basal_ = 0.329 + (0.341)(HW); *F* = 81.6, df = 1, 111, *P* < 0.001, (2) *L*_total_ = 0.0981 + (0.615)(HW); *F* = 48.1, df = 1, 111, *P* < 0.001; (3) *V*_basal_ = 0.0422 + (0.279)(HW); *F* = 56.4, df = 1, 111, *P* < 0.001, and (4) *V*_total_ = − 0.0167 + (0.374)(HW); *F* = 56.4, df = 1, 111, *P* < 0.001. Mean ± SE WW values are superimposed above the bars (sample sizes for means range from 52 to 61).

To test the hypothesis that wing wear was correlated with oocyte size (independent of date of collection and head width), we determined which females collected after 22 June had smaller- or larger-than expected values for oocytes-size variables based on the regressions of those variables on HW. Females with positive residuals (larger than expected oocyte sizes) had significantly lower WW values than those with negative residuals for *L*_basal_ (Mann–Whitney Test, *P* < 0.01), *V*_basal_ (*P* < 0.01), and *V*_total_ (*P* < 0.001), but not *L*_total_ (*P* = 0.68) (Fig. 4). These results are reflected in the overall correlations between the oocyte variables and WW which were significant and negative for *L*_basal_ (Spearman Correlation, *r* = − 0.22, *P* < 0.05), *V*_basal_ (*r* = − 0.27, *P* < 0.01), and *V*_total_ (*r* = − 0.27, *P* < 0.01), but not *L*_total_ (*r* = − 0.14, *P* = 0.14). Thus, although we observed considerable variation among females, and those with high WW scores sometimes produced large oocytes, the more wing-worn females tended to have smaller oocytes than would be predicted from their body sizes.

### Pollen counts

Of the 2,000 pollen grains identified, 100 from each of 10 females from 22 June and 10 from 3 August, all but one was from alfalfa. That single grain appeared to be from a plant of the family Asteraceae. After identifying the 100 grains from each female, we scanned the whole sample on each slide and found no other non-alfalfa pollen.

## Discussion

### Seasonal trends in wing wear

We documented a steady decline in wing condition over the first 3–4 weeks of each nesting season at both sites, with some females having wings tattered along their entire distal margins by the second or third week following bee release. Many late-season females had deep excisions in their wings. Based on mark-recapture observations of individual bees, wing wear of bumble bees (Foster & Cartar, 2011) and honey bees (Higginson & Barnard, 2004) accelerates with age. The lack of an accelerating, or even a steadily increasing, seasonal trend in wing wear for *M. rotundata* in our data could have several causes. First, unlike the studies of the social bees, we did not score WW on the same bees each week. If *M. rotundata* females with extremely worn wings die late in the season, as occurs in bumble bees (see below), they would be absent from our samples. Second, there may be an inherent non-linear relationship between age and wing wear, in which females progress through the first three (i.e., lowest) WW classes quickly, but then more slowly through the remaining WW classes, perhaps because the open wing margin is now closer to supporting wing veins. Third, the appearance of an overlapping second generation of females with less wing wear late in the season would result in smaller mean WW values if females of both generations are mixed in samples. Based on the reappearance of females with low WW values late in the season, second generation females did not show up in the MT samples until the seventh (2012) or eighth week (2011) following the initial release of the bees. Based both on WW values and the appearance of emergence holes in nests, they appeared at the UT site earlier, perhaps by weeks four and five. The great variability of wing wear in *M. rotundata*, even prior to emergence of a second generation, suggests that it is a weak predictor of mean calendar age, as is the case for the solitary bees *Anthidium manicatum* (L.) (Mueller & Wolf-Mueller, 1993), *Dieunomia triangulifera* (Vachal) (Wuellner, 1999), and *Euglossa* spp. (López-Uribe, Oi & Del Lama, 2008).

Rapid accumulation of wing wear in *M. rotundata* is expected from the high level of flight activity of nesting females. Klostermeyer & Gerber (1969) estimated that completion of a single nest cell involved an average of 15 leaf-collecting trips and 17 pollen- and nectar-foraging trips, and later estimated that females typically provision 12–16 cells during their lives (Gerber & Klostermeyer, 1972). Szabo & Smith (1972) reported that females visited an average of 7.5 flowers per foraging trip at air temperatures of 17–21 °C, and 17.8 per trip at 21–25 °C. Based on these combined estimates, females would make 384–512 total trips during their adult lives, depending on cell number and temperature, visiting thousands of flowers in the process. This may be an underestimate, because some females are known to provision >25 cells in a lifetime (T Pitts-Singer, 2011, unpublished data). The values for the variables leading to these estimates are certainly time- and site-specific, but they illustrate the amount of time females spend foraging. To this, we can add the unknown number of visits to flowers to collect nectar and pollen for self-maintenance. Although females probably forage near nests when flowers are available during the peak of alfalfa bloom, *M. rotundata* females sometimes forage hundreds of meters from nests (Tepedino, 1983; St. Amand, Skinner & Peaden, 2000). Finally, it is also possible that females sustain wing wear when (1) fighting with nest-intruding females over possession of tunnels, (2) colliding with other bees in crowded commercial bee shelters, (3) interacting with males during attempted matings, and (4) moving about in the confines of nest tunnels, which are often barely wider than their bodies (O’Neill et al., 2010).

Insight into the potential causes and consequences of wing wear in *M. rotundata* is gained from studies of other bees. In worker bumble bees, variation in wing area lost was related not to flight frequency or time spent in flight, but to collisions of wings with vegetation during foraging (Foster & Cartar, 2011). Greater degrees of wing wear increased mortality rates (Cartar, 1992) and influenced the choice of flower patches visited (Foster & Cartar, 2010), but had no effect on pollen load sizes, the duration of foraging trips (Cartar, 1992), or the energetic cost of flight (Hedenström, Ellington & Wolf, 2001). Rodd, Plowright & Owen (1980) suggest that the rapid increase in mortality rates of *Bombus* workers after two weeks of adult life is due to accumulated wing damage that hinders their ability to escape predators. In honey bees (*Apis mellifera* L.), workers with greater wing wear tended to accept smaller, lower-quality inflorescences, leading to reduced foraging efficiency (Higginson & Barnard, 2004). Similarly, an experimentally-induced reduction in wing area significantly decreases the rate of food delivery and increases mortality of worker honey bees (Dukas & Dukas, 2011). Overall, this brief review suggests that wing wear in *M. rotundata* might also be linked to foraging success, with consequences for the sustainability of nesting performance as the summer progresses. More direct experimental tests of this hypothesis are required, but such tests also need to take into account the possibility that declining performance of wing-worn females late in the season is also related to the smaller body lipid reserves and floral resource availability. In addition, there is also the question of whether the fitness consequences of declining flight performance related to wing wear are similar in social and solitary bees. In eusocial bees, older, physically-worn workers are replaced by younger bees as the season progresses, but there is no such compensating mechanism for solitary bees. One study of a solitary bee *Ceratina calcarata* showed that females with greater wing wear produced smaller clutches and smaller overall masses of adult offspring, and that wing wear appeared to be the primary correlate of declining levels of parental investment across the nesting season (Rehan & Richards, 2010).

### Seasonal trends in lipid content

In a previous study of adults active in the field in 2006 and 2007 at the same MT site in this study, female *M. rotundata* reared under controlled laboratory conditions from early fall to early summer emerged with a mean proportion body lipids of 15–18% (O’Neill et al., 2011). In the present study, the fact that the initial mean values for *P_L_* of newly-emerged field-collected females were just 7–11% could be related to several differences between the studies. First, yearly differences in floral resource quality and quantity could affect the lipid content of the bees at the time they entered diapause the previous year. Second, there might be differences in stored-lipid metabolism between bees reared under tightly-controlled laboratory conditions (O’Neill et al., 2011) and those experiencing variable on-farm rearing conditions (present study). In our earlier study, variation in temperature during pre-release rearing explained 13–27% of the variance in the proportion of body lipids of newly-emerged adults, with the optimal rearing temperatures for maintaining maximum lipid reserves being 27–29 °C. In addition, brief exposure of bees to 38 °C during the last week of adult rearing in the lab causes significant lipid loss. In the field, bees could regularly experience such spikes in temperature when cells are placed into field shelters at the time of release. In late June or early July, when females complete pupal–adult development, it would not be unusual for shelter temperatures to exceed optimal temperatures for lipid conservation. Finally, bees reared in the laboratory were frozen for lipid analysis within several minutes to several hours of their emergence as adults (O’Neill et al., 2011). In the present study, however, they were likely to be at least 1–2 days old when collected; some may have been older than that because, even in controlled laboratory conditions, females reared at the same temperature do not all emerge on the same day (O’Neill et al., 2011). Thus, during the brief interval between emergence and collection, females in the present study may have already converted a considerable portion of their fat-body lipids to proteins destined for egg production (Sihag, 1985; Sihag, 1986). In the halictid bee *Lasioglossum malachurum*, a species that overwinters as adults, depletion of fat reserves during solitary nest-founding is rapid and more lipids are lost during that period than during hibernation (Weissel et al., 2012).

Whatever the reason for the initial *P_L_* values, our results indicate that lipid content in adult females declines rapidly after field release and then stabilizes at a lower level later in the season. Some of the temporal variation we observed after the initial decline is likely due to emergence of a second generation that introduced individuals with greater lipid content into later samples. In the UT samples, the significant mid-season increase in lipid content on 18 July (i.e., JD 200), which was about one month after bee release, corresponded to an influx of newly-emerged females with lower WW values. Apparently, as these second-generation females aged, mean *P_L_* values again declined one week later. Examining the trend of *P_L_* as function of wing wear (as a measure of physiological age) rather than date, removed much of the temporal fluctuation probably because it removed the influence of second-generation females on the trend.

Because we extracted lipids in bulk, we do not know what proportion of lipids remaining after the initial decline were present in fat bodies, where they could act as nutrient reserves. Lipids extracted from tissues not involved in lipid storage likely create a baseline value to lipid content below which living females cannot decline, even after most fat body lipids have been depleted. Thus, as with wing wear, females whose lipid levels dropped below a critical level may have disappeared from the population. In addition, an unknown amount of lipids in the samples might have been derived from pollen in the guts of females so its contribution deserves further analysis. Alfalfa pollen, which constituted almost all of what we identified in dissected females, contains about 8.5% lipids (Roulston & Cane, 2002). Alfalfa pollen is typically most of what females collect when nesting in commercial alfalfa fields in Montana (O’Neill et al., 2004; O’Neill & O’Neill, 2010). The volume of pollen in the guts of females is unknown, but the estimated lipid content of an average pollen load at a time when alfalfa is the main source of pollen is just 0.06 mm^3^ (O’Neill & O’Neill, 2010). Based on qualitative assessment of pollen loads within the gut, however, we found no tendency for females to increase pollen consumption as their lipid content dropped. In fact, pollen consumption may have declined because all females collected on 22 June had crops containing large masses of pollen, whereas after that date most had very little pollen in their crops even when their midguts were full of pollen. Richards (1994) reported that 100% of *M. rotundata* females had pollen in their crops, but noted only its presence or absence, so his results are not comparable to ours. We should also re-emphasize that lab-reared females without access to pollen had even higher *P_L_* values (O’Neill et al., 2011) than the field-collected females in this study that carried pollen in their guts.

At present, it seems clear that females used up a considerable proportion of their body lipids soon after emergence, and it is reasonable to assume that this resulted mainly from fat body materials being converted to materials used to make oocytes (Sihag, 1985; Sihag, 1986; Klowden, 2002; Arrese & Soulages, 2010). A more detailed analysis of the anatomical sources of lipids extracted from females and the types of lipids extracted at different times of the year would be needed to further explore the significance of our results. There is also the question of the significance of the decline in fat body lipids for aspects of female reproductive performance. First, flight metabolism of bees is thought to be fueled by carbohydrates derived from nectar and stored in the crop rather by nutrients mobilized from fat bodies (Suarez et al., 2005). Second, fat body lipids accumulated during larval development are important sources of nutrients for egg production and adult maintenance for insects in general (Pan, Bell & Telfer, 1969; Keely, 1985; Arrese & Soulages, 2010). Bees, however, have to consume pollen to initiate oogenesis (see below), so some of the materials for oocyte production may come from the adult diet. Nevertheless, as in *Lasioglossum malachurum* (Weissel et al., 2012), depletion of lipid levels in *M. rotundata* continues during the early part of the season despite consumption of pollen soon after adult emergence.

### Seasonal trends in oocyte size

In solitary nest-provisioning Hymenoptera, eggs are larger relative to body size than they are in related parasitoid and social species, and each female lays relatively few eggs in her lifetime (Iwata, 1955; Iwata, 1960; Iwata & Sakagami, 1966; O’Neill, 2001; Rozen, 2003). Such large eggs require substantial investment from females. Our data indicate that oocyte size varied with both female size (O’Neill, Delphia & O’Neill, 2014) and collection date (present study). These results are consistent with several of Richards’ (1994) conclusions concerning seasonal trends in oocyte development in *M. rotundata*. First, females in his samples did not fully mature oocytes until the second week after eclosion; one of us (T Pitts-Singer, unpublished data), nevertheless, has observed females ovipositing just two days after emergence. *Megachile rotundata* females lay eggs only when a cell is fully provisioned, so newly-emerged females can make progress towards constructing and provisioning their first nest cells before they are capable of laying eggs. Second, although we were unable to collect females beyond ∼6 weeks past adult emergence, oocyte size showed evidence of a decline by 3 August.

The pollen masses seen in guts of recently-released females are consistent with experimental evidence that pollen consumption is a prerequisite for oocyte development. Richards (1994) showed that female *M. rotundata* fed honey, but lacking access to pollen, failed to initiate vitellogenesis. In fact, the broad taxonomic occurrence of a link between pollen consumption by newly-emerged adult female bees and their schedule of egg development suggests that it is an evolutionarily-ancient trait in apiform Apoidea. Adult consumption of pollen is known to be necessary for the initiation of vitellogenesis and oocyte development in bees of the families Apidae (Human et al., 2007), Halictidae (Bell, 1973; Wuellner, 1999), and Megachilidae (Sihag, 1986; Richards, 1994); bees of these three families are estimated to have diverged from one another 90–100 million years ago during the Cretaceous (Brady, Larkin & Danforth, 2009). Sihag (1986) also demonstrated that the inclusion of protein, rather than just carbohydrates, in an artificial diet was required for vitellogenesis in three species of south Asian *Megachile* managed for alfalfa pollination. Females of two of the species, which were multivoltine (Sihag, 1983), exhibited their highest rates of oocyte development when they emerged at the time of year when alfalfa flowers were most abundant (Sihag, 1986). At that time, the average delay between adult female emergence and first oviposition was slightly longer than four days in both species. On a natural diet of alfalfa pollen and nectar, vitellogenesis commenced in the second day following emergence and the first oocytes to develop chorions appeared on day four. In *Megachile flavipes* Spinola, vitellogenins that eventually provide proteins for eggs appear in hemolymph on the second day after emergence (Sihag, 1985).

Older *M. rotundata* females apparently reach an age when they begin to produce smaller oocytes. Richards (1994) followed a population for ∼9 weeks after emergence, and recorded a steady decline in basal oocyte volume after ∼5 weeks. Because we could follow bees only until the seventh week after emergence, we observed only the initiation of that decline. The relationship of diminishing oocyte size with advancing physiological age was substantiated by the negative correlations we observed between wing wear and oocyte size. Greater degrees of wing wear are associated with declining clutch size and total brood mass in the bee *Ceratina calcarata* (Rehan & Richards, 2010), and with fewer and smaller mature oocytes in the brood parasitic apoid wasp *Stizoides renicinctus* (O’Neill & Pearce, 2007). Egg size also declines with maternal age in honey bees (Al-Lawati & Bienefeld, 2009).

## Conclusions

Even when nesting in commercial alfalfa fields with high floral densities, *M. rotundata* females face seasonally-dynamic environments. First, higher field temperatures result in greater prevalence of a condition referred to as “pollen ball,” that is cells in which the pollen masses are not consumed and in which no bee offspring develop (Pitts-Singer & James, 2008). Second, the prevalences of chalkbrood disease and pteromalid wasp parasitism change seasonally (O’Neill, 2004). Third, floral resource quality and quantity declines as alfalfa senesces (Strickler & Freitas, 1999; see also Kim & Thorp, 2001; Pitts-Singer, 2013a), with the result that pollen types used change seasonally (O’Neill & O’Neill, 2010). Lower resource levels for caged females in experimental studies resulted in smaller offspring size and fewer offspring per female (Peterson & Roitberg, 2006). Seasonal declines in investment per offspring have also been documented for the megachilid bee *Osmia lignaria* (Torchio & Tepedino, 1980), though not for the megachilid *Hoplitis anthocopoides* (Schenk) (Strickler, 1982). Fourth, in nest shelters provided for *M. rotundata*, the number of available nest tunnels may decline later in the summer and higher ratios of females to available nest sites have been associated with lower offspring production (Mayer, 1994; but see Pitts-Singer, 2013a).

The results of this and other studies (Richards, 1994) make it clear that, besides the environmental factors discussed above, seasonal changes in the condition of the females themselves might also affect the temporal consistency of reproductive performance. Females experience a steady increase in wing wear that could affect their flight efficiency and resource choices. Soon after emergence, they face a rapid drop in lipid stores that can be ameliorated only by feeding on pollen, and any pollen collected for personal consumption cannot be used for nest provisioning. Lastly, relative oocyte size starts to decline within six weeks of emergence, continues through the remainder of the summer (Richards, 1994), and is perhaps related to declining lipid reserves.

During management of *M. rotundata* for pollination services (Pitts-Singer & Cane, 2011), not all factors that affect female reproductive success can be controlled (e.g., weather, wing wear). However, certain management practices have the potential to impact adult female reproductive performance in ways related to our findings. First, because females vary considerably in the amount of lipids they carry into adult life and then rapidly deplete these reserves upon emergence, it is important to rear bees at temperatures that enhance conservation of lipid reserves sequestered during larval development (Pitts-Singer & James, 2009; O’Neill et al., 2011). Second, because females require a source of pollen following emergence, in order to stimulate oogenesis and supplement nutrients no longer supplied by fat body reserves, care must be taken to release females at a time when sufficient bloom is present to support female pollen requirements (as has also been recommended by Richards, 1994). The date of commencement of alfalfa bloom is influenced by the vagaries of weather. However, manipulation of pre-release rearing temperatures can be used to slow bee development if bloom is delayed (Yocum et al., 2010), not only to ensure adequate pollen resources for females, but to enhance levels of pollination (Bosch & Kemp, 2005). Nevetheless, although seasonal changes in condition have been documented, the degree to which these changes affect physiological performance and fitness remains to be fully explored.

## Supplemental Information

10.7717/peerj.930/supp-1 Supplemental Information 1 Supplementary Data
