# Supplementary material for: Seasonal trends in the condition of nesting females of a solitary bee: wing wear, lipid content, and oocyte size
Source: PeerJ. 2015 May 7;3:e930. doi: 10.7717/peerj.930 (PMC4435504; doi:10.7717/peerj.930)
Supplement: Supplemental Information 1 [file peerj-03-930-s001.pdf]

Seasonal Trends in the Condition of Nesting Females of a Solitary Bee: Wing Wear, Lipid Content, and Oocyte (K.M. O'Neill, C.M. Delphia, T.L. Pitts-Singer).

Supplementary Table 1. Mean wing wear index scores (WW) and proportion body lipids (P_L) for females collected on different dates in Montana in 2011. Females for each date were given unique identifier numbers. See Materials and Methods for explanation.

	7 July		21 July		28 July		11 August		25 August	
Female	WW	P_L	WW	P_L	WW	P_L	WW	P_L	WW	P_L
1	0.0	0.050	1.0	0.073	3.0	0.046	2.5	0.047	4.0	0.053
2	0.0	0.086	2.0	0.053	3.5	0.075	3.0	0.062	3.5	0.054
3	0.0	0.058	1.5	0.061	3.0	0.032	4.0	0.044	3.5	0.057
4	0.0	0.058	3.0	0.044	2.5	0.067	3.0	0.105	4.0	0.051
5	0.0	0.048	2.0	0.062	3.5	0.018	4.0	0.046	2.0	0.056
6	0.0	0.106	1.5	0.053	4.0	0.038	3.0	0.046	4.0	0.037
7	0.0	0.108	0.5	0.078	4.0	0.038	4.0	0.056	4.0	0.060
8	0.0	0.093	2.0	0.062	6.0	0.048	2.5	0.050	2.0	0.066
9	0.5	0.041	2.0	0.051	3.0	0.048	3.0	0.029	2.0	0.059
10	0.0	0.099	2.0	0.066	3.5	0.047	3.5	0.036	1.5	0.027
11	0.0	0.065	2.0	0.066	3.0	0.092	3.5	0.054	4.0	0.016
12	0.0	0.041	2.0	0.068	3.0	0.028	3.5	0.038	4.5	0.049
13	0.0	0.104	2.0	0.069	3.5	0.047	4.0	0.056	2.0	0.046
14	0.5	0.042	4.0	0.046	3.5	0.039	2.5	0.049	4.0	0.038
15	0.0	0.083	2.0	0.055	4.5	0.039	3.0	0.038	4.0	0.071
16	0.5	0.048	1.0	0.064	5.0	0.046	3.0	0.050	4.0	0.057
17	0.0	0.101	2.5	0.059	3.5	0.035	3.5	0.024	4.5	0.055
18	1.0	0.078	1.5	0.047	5.0	0.064	2.5	0.041	2.5	0.055
19	0.0	0.147	2.0	0.049	4.0	0.052	4.0	0.036	3.0	0.056
20	0.0	0.108	3.0	0.055	3.5	0.058	4.0	0.047	3.5	0.055
21	0.0	0.052	3.0	0.050	3.0	0.057	3.5	0.048	4.0	0.034
22	0.0	0.037	2.0	0.074	3.0	0.021	2.5	0.032	4.0	0.039
23	0.0	0.078	2.0	0.067	3.0	0.051	5.0	0.049	4.0	0.047
24	0.0	0.111	3.5	0.038	3.0	0.060	4.0	0.045	4.5	0.086
25	0.0	0.033	2.0	0.059	3.0	0.053	4.0	0.033	3.5	0.051

26	0.0	0.064	3.0	0.051	2.0	0.051	4.5	0.057	2.5	0.054
27	0.5	0.070	3.5	0.050	3.0	0.027	3.5	0.034	3.5	0.053
28	0.0	0.050	4.0	0.058	2.5	0.053	4.0	0.043	2.0	0.071
29	0.0	0.080	4.0	0.033	2.0	0.053	2.5	0.047	2.5	0.048
30	0.0	0.109	3.5	0.058	2.0	0.008	3.5	0.039	4.0	0.071
31	0.0	0.126	2.5	0.059	1.0	0.039	3.5	0.057	1.5	0.052
32	0.0	0.047	5.0	0.051	3.0	0.059	3.5	0.033	4.0	0.042
33	0.0	0.119	3.0	0.054	3.0	0.034	3.5	0.030	4.0	0.079
34	0.0	0.099	4.0	0.034	2.0	0.058	4.0	0.050	5.5	0.065
35	0.0	0.075	2.0	0.074	2.0	0.040	4.0	0.042	3.5	0.047
36	0.0	0.057	3.5	0.047	2.0	0.041	4.0	0.043	3.5	0.038
37	0.0	0.081	2.5	0.023	4.0	0.056	6.0	0.042	2.0	0.056
38	0.0	0.099	3.0	0.043	3.0	0.029	4.5	0.046	4.0	0.049
39	0.0	0.047	2.0	0.047	3.0	0.050	2.5	0.024	4.0	0.060
40	0.0	0.052	2.0	0.042	2.0	0.047	3.0	0.033	4.0	0.029
41	0.0	0.095	4.0	0.053	2.0	0.050	2.0	0.049	1.5	0.058
42	0.0	0.043	3.0	0.031	2.0	0.040	3.0	0.058	4.0	0.062
43	0.5	0.024	4.5	0.039	1.0	0.074	4.0	0.041	6.0	0.036
44	1.0	0.075	4.0	0.088	1.0	0.036	3.5	0.051	6.0	0.039
45	0.0	0.116	4.0	0.035	2.0	0.050	3.5	0.041	4.5	0.050
46	0.0	0.060	3.5	0.079	0.5	0.063	3.0	0.067	4.0	0.056
47	0.0	0.128	4.5	0.063	3.0	0.060	3.5	0.038	0.5	0.057
48	0.0	0.038	4.0	0.057	2.5	0.044	3.0	0.043	2.0	0.034
49	0.0	0.101	4.5	0.045	1.0	0.063	4.0	0.054	5.0	0.062
50	0.0	0.044	3.5	0.053	1.5	0.057	2.0	0.061	5.0	0.047

Supplementary Table 2. Same information as in Supplementary Table 1, but for bees collected in Montana in 2012.

	22 June		29 June		6 July		13 July		20 July		27 July		3 August	
Female	WW	P_L	WW	P_L	WW	P_L	WW	P_L	WW	P_L	WW	P_L	WW	P_L
1	0.0	0.088	1.0	0.043	1.0	0.055	4.5	0.045	3.0	0.007	4.5	0.016	5.0	0.037
2	0.0	0.108	1.5	0.044	2.0	0.046	2.5	0.040	4.0	0.043	4.0	0.040	4.5	0.021
3	0.0	0.070	1.5	0.056	2.0	0.044	2.0	0.039	4.5	0.018	3.0	0.048	3.0	0.037
4	0.0	0.053	2.0	0.049	2.0	0.064	4.0	0.051	4.0	0.067	4.0	0.029	4.5	0.044
5	0.0	0.093	1.5	0.052	2.5	0.016	4.0	0.041	3.5	0.047	2.5	0.039	5.5	0.035
6	0.0	0.054	1.5	0.037	2.0	0.037	4.0	0.032	4.0	0.034	3.0	0.091	4.0	0.062
7	0.0	0.084	3.0	0.046	0.5	0.053	2.5	0.050	3.5	0.043	2.5	0.045	0.5	0.156
8	0.0	0.060	2.0	0.030	2.0	0.054	2.5	0.045	4.5	0.047	4.0	0.034	4.0	0.047
9	0.0	0.059	2.0	0.021	3.0	0.031	3.0	0.022	4.0	0.039	4.0	0.017	5.0	0.062
10	0.5	0.029	2.0	0.036	2.0	0.050	4.0	0.023	4.0	0.061	3.5	0.039	5.5	0.008
11	0.0	0.109	3.0	0.036	2.0	0.065	5.0	0.034	3.0	0.033	4.0	0.040	4.0	0.050
12	0.5	0.060	0.5	0.040	2.0	0.038	1.5	0.066	4.0	0.032	4.0	0.051	1.0	0.066
13	0.0	0.070	1.5	0.036	3.0	0.043	3.5	0.023	3.5	0.073	5.5	0.033	4.5	0.017
14	0.0	0.056	2.0	0.027	4.0	0.037	2.5	0.056	4.5	0.041	4.5	0.051	4.0	0.050
15	0.0	0.048	0.0	0.055	2.0	0.040	3.0	0.061	3.0	0.053	4.5	0.015	5.5	0.038
16	0.0	0.063	2.0	0.038	2.5	0.039	3.5	0.044	4.0	0.036	4.0	0.037	0.0	0.158
17	0.0	0.046	2.0	0.051	2.0	0.048	3.5	0.042	4.0	0.040	4.0	0.057	3.0	0.027
18	0.0	0.072	1.0	0.049	2.0	0.031	2.0	0.043	3.0	0.027	4.0	0.042	0.5	0.070
19	0.0	0.022	1.0	0.048	2.0	0.049	3.5	0.053	3.5	0.044	3.5	0.044	6.0	0.029
20	0.5	0.026	1.5	0.042	2.0	0.035	4.0	0.041	3.0	0.041	4.5	0.026	4.0	0.014
21	0.0	0.016	1.0	0.037	2.0	0.023	2.5	0.050	4.0	0.055	4.0	0.050	0.0	0.050
22	0.0	0.112	0.5	0.043	2.5	0.022	3.0	0.047	2.5	0.036	3.5	0.012	4.5	0.029
23	0.5	0.044	2.0	0.061	1.5	0.034	1.5	0.047	2.0	0.042	4.5	0.069	2.5	0.043
24	0.5	0.053	1.0	0.034	2.0	0.045	4.5	0.061	4.0	0.040	4.5	0.053	3.5	0.007
25	0.0	0.053	1.0	0.051	3.0	0.046	4.5	0.043	3.5	0.065	5.0	0.033	4.0	0.038
26	0.5	0.056	2.0	0.036	1.0	0.031	1.5	0.040	4.0	0.041	4.0	0.029	4.5	0.033
27	0.0	0.111	1.5	0.042	2.5	0.053	2.5	0.035	4.0	0.054	3.0	0.029	4.5	0.048
28	0.5	0.091	2.0	0.036	2.0	0.044	2.5	0.049	4.5	0.064	4.0	0.027	3.5	0.043
29	0.5	0.068	1.5	0.026	2.0	0.030	4.5	0.034	2.5	0.086	4.5	0.057	4.0	0.071
30	1.0	0.117	2.0	0.007	1.0	0.045	3.0	0.024	3.0	0.056	5.5	0.028	3.5	0.053

Supplementary Table 3. Same information as in Supplementary Table 1, but for bees collected in Utah in 2012.

	20 June		27 June		3 July		11 July		18 July		25 July	
Female	WW	P_L	WW	P_L	WW	P_L	WW	P_L	WW	P_L	WW	P_L
1	1.0	0.126	2.0	0.049	0.0	0.069	5.5	0.048	3.0	0.068	5.5	0.008
2	0.5	0.123	2.0	0.095	1.0	0.052	5.5	0.056	4.0	0.047	4.5	0.064
3	0.0	0.106	0.5	0.073	1.5	0.073	4.0	0.061	3.5	0.041	5.0	0.055
4	1.5	0.062	2.5	0.061	4.0	0.057	2.5	0.067	1.5	0.127	4.0	0.048
5	1.5	0.056	1.5	0.067	3.0	0.044	3.0	0.052	1.5	0.082	5.5	0.063
6	0.0	0.107	0.5	0.084	3.0	0.073	4.0	0.071	3.5	0.040	3.5	0.053
7	0.0	0.105	1.0	0.074	0.0	0.048	3.0	0.062	1.0	0.061	4.5	0.087
8	0.0	0.125	2.0	0.061	0.0	0.071	0.5	0.102	2.0	0.070	4.0	0.040
9	0.0	0.074	3.0	0.066	0.5	0.036	5.5	0.056	0.5	0.099	6.0	0.059
10	0.0	0.186	2.5	0.056	2.5	0.052	3.0	0.050	4.5	0.056	4.5	0.020
11	0.5	0.160	2.5	0.070	1.0	0.056	4.5	0.069	0.0	0.100	5.0	0.050
12	0.0	0.176	2.0	0.044	3.0	0.050	3.5	0.066	5.0	0.036	4.0	0.036
13	0.0	0.078	2.5	0.071	1.5	0.042	2.0	0.072	3.5	0.042	4.0	0.047
14	0.5	0.126	0.0	0.116	1.0	0.083	3.0	0.059	0.0	0.103	5.0	0.046
15	0.5	0.090	2.5	0.047	0.0	0.110	5.0	0.068	0.5	0.097	4.0	0.057
16	0.0	0.112	1.5	0.083	1.0	0.047	4.5	0.037	6.0	0.054	3.0	0.046
17	0.5	0.081	2.0	0.078	0.0	0.050	3.0	0.060	0.5	0.103	4.0	0.081
18	0.5	0.197	2.0	0.068	0.5	0.088	4.0	0.071	1.5	0.064	4.5	0.068
19	0.0	0.097	0.5	0.068	0.0	0.141	3.0	0.063	0.5	0.085	4.0	0.056
20	1.0	0.058	2.0	0.059	3.0	0.068	3.0	0.050	6.0	0.055	6.0	0.067
21	0.5	0.135	3.0	0.061	4.0	0.089	4.0	0.031	0.5	0.083	4.5	0.034
22	0.5	0.076	2.0	0.051	0.5	0.051	5.0	0.046	4.0	0.057	4.0	0.058
23	0.5	0.082	2.0	0.043	0.0	0.096	4.0	0.054	1.5	0.062	3.5	0.066
24	0.5	0.157	2.5	0.039	0.0	0.076	4.0	0.044	4.5	0.053	3.0	0.026
25	1.0	0.105	2.0	0.050	2.0	0.057	4.5	0.071	1.0	0.058	6.0	0.046
26	0.0	0.163	1.0	0.085	0.5	0.041	1.0	0.058	0.0	0.126	4.0	0.037
27	0.0	0.194	1.0	0.079	1.0	0.077	4.0	0.060	1.0	0.121	4.5	0.078
28	1.0	0.055	2.0	0.063	1.5	0.074	4.5	0.054	0.0	0.079	6.0	0.045
29	0.5	0.034	2.5	0.065	3.5	0.069	2.5	0.063	1.0	0.118	3.5	0.066
30	1.0	0.051	2.0	0.077	0.5	0.058	4.5	0.024	1.5	0.063	4.0	0.046

Supplementary Table 3 (continued)

	1 August		8 August		15 August		22 August	
Female	WW	P_L	WW	P_L	WW	P_L	WW	P_L
1	1.0	0.087	2.0	0.066	2.5	0.055	2.0	0.062
2	0.0	0.053	1.5	0.050	3.5	0.056	1.0	0.080
3	4.0	0.080	3.0	0.062	3.0	0.068	3.0	0.066
4	3.0	0.032	0.5	0.080	0.0	0.055	2.5	0.036
5	6.0	0.062	2.5	0.035	2.0	0.057	2.0	0.054
6	6.0	0.062	0.0	0.078	3.0	0.043	2.0	0.049
7	5.5	0.047	2.5	0.087	4.0	0.046	3.5	0.069
8	4.0	0.056	0.0	0.062	4.0	0.087	2.0	0.061
9	0.5	0.050	2.0	0.067	2.0	0.056	2.0	0.068
10	4.0	0.038	0.5	0.075	4.0	0.057	3.0	0.049
11	4.0	0.063	0.0	0.060	1.5	0.053	3.0	0.076
12	6.0	0.049	1.5	0.058	1.0	0.064	1.5	0.091
13	4.0	0.050	1.0	0.090	1.0	0.096	2.5	0.081
14	4.0	0.058	0.5	0.043	1.5	0.056	3.0	0.083
15	4.0	0.023	1.0	0.062	1.0	0.066	4.0	0.069
16	6.0	0.041	1.5	0.080	2.5	0.064	1.0	0.054
17	2.0	0.066	1.0	0.050	0.5	0.036	1.5	0.026
18	4.5	0.038	2.5	0.042	2.5	0.041	1.5	0.077
19	2.0	0.063	4.0	0.067	3.5	0.039	2.5	0.049
20	1.5	0.106	1.5	0.054	6.0	0.053	3.0	0.054
21	1.0	0.090	1.0	0.096	2.0	0.077	3.0	0.032
22	3.0	0.049	2.0	0.091	3.0	0.063	3.0	0.052
23	0.0	0.080	4.0	0.055	3.0	0.081	3.5	0.069
24	6.0	0.051	1.5	0.045	3.0	0.066	4.5	0.053
25	1.5	0.059	1.0	0.069	4.0	0.059	3.5	0.053
26	1.5	0.043	1.0	0.074	3.0	0.086	2.5	0.065
27	5.0	0.053	0.0	0.068	2.0	0.083	0.5	0.120
28	4.5	0.039	4.0	0.060	2.0	0.068	2.5	0.059
29	2.0	0.063	2.0	0.040	2.0	0.062	2.0	0.076
30	1.0	0.094	1.0	0.053	3.0	0.074	2.0	0.063

Supplementary Table 4. Head widths (in mm), wing wear scores (WW), and lengths and widths (mm) of three largest oocytes from females collected on five dates in Montana in 2012. For purposes of calculating oocyte volumes, basal oocytes were recorded as being prolate-spheroid shaped (PS) or sausage shaped (SS); oocytes 2 and 3 are not ranked by size. Females for each date were given unique identifier numbers. See Materials and Methods for explanation.

	Collection	Female	Female	Basal oocyte		Oocyte #2		Oocyte #3		Basal oocyte
Female	Date	HW	WW	L	W	L	W	L	W	shape
1	22 June	3.15	1	0.75	0.25	0.50	0.20	0.30	0.15	PS
2	22 June	2.80	0	0.75	0.20	0.40	0.15	0.25	0.10	PS
3	22 June	3.10	0	1.05	0.35	0.60	0.20	0.25	0.15	PS
4	22 June	2.75	1	0.45	0.20	0.20	0.20	0.35	0.10	PS
5	22 June	3.25	0	0.70	0.25	0.25	0.25	0.40	0.15	PS
6	22 June	3.30	0	1.05	0.20	0.20	0.20	0.60	0.15	PS
7	22 June	3.05	1	0.70	0.15	0.15	0.15	0.55	0.15	PS
8	22 June	3.10	0	1.00	0.30	0.15	0.15	0.35	0.15	PS
9	22 June	2.85	0	0.85	0.25	0.20	0.20	0.25	0.10	PS
10	22 June	3.40	1	1.25	0.40	0.20	0.20	0.65	0.15	PS
11	22 June	3.30	0	1.20	0.30	0.75	0.30	0.50	0.20	SS
12	22 June	3.10	0	1.00	0.30	0.35	0.20	0.35	0.10	SS
13	22 June	3.15	0	0.80	0.35	0.50	0.15	0.35	0.10	SS
14	22 June	3.40	0	1.15	0.30	0.90	0.25	0.80	0.25	SS
15	22 June	3.35	0	1.45	0.40	0.65	0.25	0.65	0.10	SS
16	22 June	3.00	0	1.10	0.40	0.60	0.30	0.30	0.10	SS
17	22 June	3.15	0	1.20	0.40	0.90	0.25	0.60	0.25	SS
18	22 June	3.10	0	1.05	0.15	0.15	0.15	0.30	0.10	SS
19	22 June	3.40	0	1.60	0.40	0.40	0.40	0.50	0.25	SS
20	22 June	3.25	0	1.50	0.30	0.30	0.30	0.55	0.30	SS
21	22 June	3.05	0	1.05	0.25	0.25	0.25	0.50	0.10	SS
22	22 June	3.25	0	1.15	0.20	0.20	0.20	0.40	0.10	SS
23	22 June	3.20	0	1.30	0.20	0.20	0.20	0.50	0.20	SS
24	22 June	3.35	0	1.40	0.40	0.25	0.25	0.65	0.15	SS
25	22 June	3.35	1	1.50	0.45	0.35	0.35	0.35	0.20	SS
1	29 June	3.40	2	2.35	0.70	1.80	0.55	1.40	0.55	SS
2	29 June	2.90	4	1.80	0.60	1.35	0.40	1.00	0.45	SS

3	29 June	3.30	2	2.25	0.55	1.60	0.45	0.80	0.35	SS
4	29 June	2.90	1	1.25	0.50	0.40	0.25	0.30	0.20	SS
5	29 June	3.35	2	1.70	0.55	0.60	0.45	0.60	0.30	SS
6	29 June	2.90	1	1.45	0.50	0.90	0.40	0.55	0.30	SS
7	29 June	3.40	0	1.85	0.55	1.85	0.45	1.25	0.50	SS
8	29 June	3.50	1	2.15	0.60	1.55	0.50	1.60	0.50	SS
9	29 June	3.15	1	2.10	0.55	1.50	0.50	0.85	0.45	SS
10	29 June	3.20	3	1.70	0.55	1.60	0.50	1.00	0.35	SS
11	29 June	3.30	1	1.95	0.50	0.40	0.40	1.00	0.45	SS
12	29 June	3.35	1	1.55	0.55	0.40	0.40	1.00	0.45	SS
13	29 June	3.30	1	2.30	0.60	0.50	0.50	0.75	0.25	SS
14	29 June	3.45	2	2.15	0.60	0.50	0.50	1.00	0.35	SS
15	29 June	3.10	0	1.95	0.55	0.50	0.50	0.80	0.40	SS
16	29 June	3.15	2	2.00	0.55	0.50	0.50	0.90	0.50	SS
17	29 June	3.20	2	2.25	0.55	0.45	0.45	0.80	0.35	SS
18	29 June	3.40	1	2.25	0.65	0.45	0.45	0.80	0.30	SS
19	29 June	3.35	1	2.25	0.55	0.50	0.50	1.05	0.40	SS
20	29 June	3.05	0	1.80	0.55	0.45	0.45	0.60	0.35	SS
21	29 June	3.15	1	1.85	0.50	0.40	0.40	0.55	0.25	SS
22	29 June	3.65	0	2.30	0.70	0.45	0.45	0.95	0.45	SS
23	29 June	3.25	1	1.90	0.55	0.45	0.45	0.70	0.35	SS
24	29 June	3.25	1	2.25	0.70	0.45	0.45	0.80	0.35	SS
25	29 June	3.15	2	1.90	0.55	0.45	0.45	0.75	0.30	SS
26	29 June	3.05	2	1.50	0.45	0.80	0.40	0.70	0.35	SS
1	6 July	3.20	0	1.80	0.50	1.65	0.50	1.50	0.50	SS
2	6 July	3.05	2	1.70	0.50	1.50	0.45	0.75	0.35	SS
3	6 July	3.20	2	1.50	0.50	1.30	0.40	0.75	0.35	SS
4	6 July	3.20	2	1.50	0.50	1.45	0.45	1.30	0.35	SS
5	6 July	3.20	1	1.65	0.55	1.10	0.45	0.60	0.20	SS
6	6 July	3.35	0	2.40	0.70	1.75	0.45	1.20	0.45	SS
7	6 July	3.30	0	2.25	0.55	1.70	0.50	1.35	0.50	SS
8	6 July	3.15	1	1.55	0.45	1.15	0.43	0.65	0.30	SS
9	6 July	3.35	4	2.10	0.50	1.60	0.50	0.75	0.50	SS
10	6 July	3.40	2	2.35	0.70	1.65	0.45	1.00	0.45	SS

11	6 July	3.30	3	1.95	0.50	1.65	0.45	1.35	0.50	SS
12	6 July	2.90	1	1.70	0.50	1.60	0.55	0.75	0.40	SS
13	6 July	3.05	1	1.75	0.45	1.05	0.35	0.55	0.25	SS
14	6 July	3.25	2	1.85	0.50	1.25	0.55	0.75	0.35	SS
15	6 July	3.50	2	1.75	0.45	1.70	0.50	1.55	0.45	SS
16	6 July	3.45	2	2.25	0.70	1.90	0.50	1.20	0.45	SS
17	6 July	3.20	5	1.75	0.50	1.55	0.50	0.95	0.45	SS
18	6 July	2.95	1	1.55	0.45	1.55	0.45	1.05	0.40	SS
19	6 July	3.35	2	2.65	0.65	2.30	0.65	1.80	0.45	SS
20	6 July	3.35	1	2.45	0.55	1.60	0.50	1.20	0.40	SS
21	6 July	3.05	1	1.80	0.50	1.55	0.45	0.85	0.45	SS
22	6 July	3.30	1	2.00	0.65	1.80	0.55	0.70	0.35	SS
23	6 July	3.45	5	1.75	0.50	1.10	0.40	0.50	0.20	SS
24	6 July	3.15	1	1.80	0.50	1.65	0.50	1.05	0.30	SS
25	6 July	3.30	2	1.55	0.50	1.30	0.50	0.75	0.25	SS
26	6 July	3.25	2	1.85	0.40	1.80	0.50	0.90	0.30	SS
27	6 July	3.25	1	1.85	0.55	1.60	0.55	1.20	0.35	SS
28	6 July	3.10	5	1.70	0.50	0.75	0.25	0.50	0.15	SS
29	6 July	3.50	3	2.40	0.60	2.00	0.50	0.85	0.40	SS
30	6 July	3.15	2	2.10	0.50	1.75	0.40	1.00	0.30	SS
31	6 July	2.80	1	1.65	0.50	1.40	0.40	0.70	0.40	SS
32	6 July	3.70	2	2.50	0.70	1.95	0.55	1.20	0.40	SS
1	20 July	3.05	2	1.55	0.55	1.15	0.35	0.50	0.25	SS
2	20 July	3.20	3	1.80	0.62	1.10	0.50	0.90	0.30	SS
3	20 July	3.15	5	2.10	0.55	1.65	0.50	0.65	0.35	SS
4	20 July	3.30	4	1.85	0.50	1.60	0.50	0.35	0.20	SS
5	20 July	3.40	4	1.65	0.45	1.65	0.45	0.50	0.25	SS
6	20 July	3.40	5	2.25	0.60	1.50	0.50	1.10	0.35	SS
7	20 July	3.25	5	1.80	0.60	1.55	0.55	0.80	0.25	SS
8	20 July	3.20	5	1.80	0.50	1.15	0.50	1.50	0.20	SS
9	20 July	3.25	4	2.10	0.60	1.35	0.30	0.55	0.25	SS
10	20 July	3.35	4	1.65	0.45	1.30	0.40	0.003	0.25	PS
11	20 July	3.10	5	1.80	0.50	1.30	0.45	0.55	0.25	SS
12	20 July	3.35	3	2.00	0.50	1.50	0.35	0.70	0.30	SS

13	20 July	3.35	4	1.95	0.45	1.85	0.45	1.40	0.30	SS
14	20 July	3.40	4	2.45	0.60	1.40	0.40	0.70	0.30	SS
15	20 July	3.00	3	1.40	0.45	1.00	0.35	0.60	0.45	SS
16	20 July	3.10	3	1.55	0.50	0.90	0.50	0.65	0.35	SS
17	20 July	3.20	3	1.95	0.55	1.75	0.55	0.80	0.45	SS
18	20 July	3.10	4	1.55	0.45	1.45	0.35	1.05	0.35	SS
19	20 July	2.95	5	1.55	0.45	0.50	0.30	0.35	0.15	SS
20	20 July	3.25	4	2.00	0.60	1.40	0.50	1.05	0.35	SS
21	20 July	3.10	6	1.65	0.50	1.00	0.40	0.55	0.25	SS
22	20 July	3.50	3	1.65	0.40	1.00	0.35	0.50	0.35	SS
23	20 July	3.30	4	1.75	0.50	0.90	0.40	0.60	0.30	SS
24	20 July	3.30	4	1.95	0.50	1.25	0.40	0.70	0.30	SS
25	20 July	3.15	3	1.40	0.50	0.75	0.35	0.45	0.20	SS
26	20 July	3.00	4	1.55	0.45	0.75	0.25	0.30	0.15	SS
27	20 July	3.05	5	1.65	0.45	0.95	0.40	0.45	0.15	SS
1	3 August	3.15	5	1.60	0.40	1.55	0.50	1.00	0.45	SS
2	3 August	3.35	5	1.85	0.55	1.00	0.50	0.50	0.20	SS
3	3 August	2.70	5	1.30	0.40	1.05	0.50	0.50	0.15	SS
4	3 August	3.30	5	1.90	0.55	1.30	0.50	0.60	0.30	SS
5	3 August	2.85	4	1.60	0.45	0.50	0.20	0.20	0.15	SS
6	3 August	2.90	4	1.25	0.45	0.70	0.25	0.45	0.15	SS
7	3 August	2.80	1	1.45	0.45	1.10	0.45	0.45	0.20	SS
8	3 August	2.50	4	0.90	0.35	0.50	0.15	0.25	0.10	SS
9	3 August	3.45	3	2.00	0.50	1.30	0.25	1.00	0.45	PS
10	3 August	2.80	4	1.20	0.50	0.93	0.60	0.45	0.20	SS
11	3 August	3.25	4	1.40	0.50	0.65	0.55	0.48	0.15	SS
12	3 August	3.40	3	1.30	0.50	0.65	0.20	0.30	0.15	PS
13	3 August	3.25	5	1.85	0.50	1.05	0.35	0.60	0.20	PS
14	3 August	3.15	3	2.10	0.50	0.95	0.50	1.00	0.45	SS
15	3 August	3.05	4	1.75	0.50	1.75	0.45	0.65	0.30	PS
16	3 August	3.35	2	2.75	0.75	1.50	0.50	1.50	0.55	PS
17	3 August	2.95	3	1.95	0.55	1.40	0.50	0.65	0.30	SS
18	3 August	3.50	3	2.25	0.70	1.40	0.75	0.85	0.40	SS
19	3 August	3.35	6	1.50	0.50	1.70	0.45	0.50	0.15	SS

20	3 August	3.05	5	1.50	0.50	1.45	0.40	0.45	0.30	SS
21	3 August	3.15	5	1.95	0.50	0.80	0.45	0.50	0.30	PS
22	3 August	3.25	5	1.95	0.60	1.65	0.70	1.30	0.60	SS
23	3 August	3.05	5	1.55	0.45	0.35	0.15	0.65	0.35	SS
24	3 August	3.20	5	1.25	0.50	0.90	0.25	0.90	0.45	SS
25	3 August	3.10	2	1.60	0.50	0.65	0.40	0.50	0.25	PS
26	3 August	3.45	6	2.25	0.65	1.50	0.40	0.25	0.15	SS
27	3 August	3.10	4	1.75	0.50	1.25	0.30	0.95	0.40	PS
28	3 August	3.45	6	2.10	0.55	1.70	0.55	1.20	0.45	SS
